# Editorial: Multi-omics in head and neck cancer: unveiling immunological biomarkers for therapy

**DOI:** 10.3389/fonc.2026.1782165

**Published:** 2026-01-23

**Authors:** Dilinaer Wusiman, Benedetta Lombardi Stocchetti, Erika Adriana Eksioglu, Stefano Cavalieri

**Affiliations:** 1 Department of Comparative Pathobiology, College of Veterinary Medicine, Purdue University, West Lafayette, IN, United States; 2 Purdue Institute for Cancer Research, Purdue University, West Lafayette, IN, United States; 3 Head and Neck Medical Oncology Department, Fondazione IRCCS Istituto Nazionale dei Tumori di Milano, Milan, Italy; 4 Moffitt Cancer Center Department of Immuno-oncology, Tampa, FL, United States; 5 Department of Oncology and Hemato-oncology, University of Milan, Milan, Italy

**Keywords:** head and neck cancer, immunological biomarkers, immunotherapy, multi-omics, tumor immune microenvironment

Head and neck cancer (HNC), encompassing a diverse group of malignancies such as head and neck squamous cell carcinoma (HNSCC), including nasopharyngeal carcinoma (NPC), remains a significant global health challenge. With over 800,000 new cases annually, HNC is characterized by high morbidity and mortality rates, often due to late diagnosis, aggressive progression, and limited therapeutic options. Traditional treatments like surgery, radiotherapy, and chemotherapy have improved survival, but recurrence and metastasis persist as major hurdles. The advent of immunotherapy, particularly immune checkpoint inhibitors, has revolutionized HNC management, yet response rates vary widely, underscoring the need for reliable biomarkers to guide personalized therapies. The rise of multi-omics, which integrates genomic, transcriptomic, proteomic, metabolomic, and microbiomic data, has opened new ways to understand the tumor and its immune environment. By combining multiple omic data layers, researchers can see how genetic and immune factors work together to shape cancer behavior and therapy outcomes. This Research *Topic “Multi-Omics in Head and Neck Cancer: Unveiling Immunological Biomarkers for Therapy”* brings together cutting-edge studies employing multi-omics approaches to identify immunological biomarkers that can guide therapeutic decision-making in HNC.

Liquid-liquid phase separation (LLPS), which generates membraneless organelles, enables the compartmentalization of macromolecules to regulate chromatin remodeling and gene expression, while also modulating cell adhesion and matrix degradation to enhance tumour invasion, and potentially influence immunotherapy responses. Zhai et al. demonstrate the transformative potential of LLPS analysis in HNSCC classification. By integrating transcriptomic data from 3,541 LLPS-related genes across 501 HNSCC patients, they identified three distinct molecular subtypes with markedly different prognoses, genomic alterations, and tumor immune microenvironment patterns. Their LLPS-related prognostic risk signature (LPRS) outperformed traditional staging systems and successfully predicted responses to immune checkpoint inhibitor therapy, while also facilitating the identification of new potential therapeutic compounds.

The predictive power of multi-omics extends to peripheral blood analysis, as demonstrated by Chihab et al., who systematically compared neoepitope prediction algorithms using samples from 11 HNSCC patients. Their comparative analysis of the current Tumor Neoantigen Selection Alliance (TESLA) pipeline and the new Identify-Prioritize-Validate (IPV) approach revealed that IPV’s use of longer peptides (20-mers) covering both CD4 and CD8 epitopes significantly outperformed TESLA’s minimal epitope approach (8-12mers) in predicting neoepitopes capable of eliciting an immune response. This finding challenges conventional paradigms in neoepitope prediction and highlights the importance of considering both HLA class I and class II restricted epitopes, a consideration often overlooked in current prediction tools that focus exclusively on CD8+ T cell responses. This work highlights IPV’s applicability for limited samples, emphasizing the need for metrics tailored to real-world immunotherapy design.

A distinctive strength of this Research Topic is its emphasis on longitudinal immune monitoring throughout treatment, especially considering the recently published result from KEYNOTE-689 and NIVOPOSTOP trials and current lack of validated immune biomarkers in this setting. One key study outlines a prospective multicenter trial aimed at defining intra-tumoral and systemic immune biomarkers in locally advanced HNC. Donaubauer et al. describe the ImmunBioKHT protocol, which enrolls 1,000 HNSCC patients and 100 controls to analyze tumor tissue, peripheral blood, and microbiome samples before and after treatments [3]. Their preliminary analysis of peripheral blood samples from 150 patients reveals dynamic immune modulations following surgery and chemoradiotherapy, with significant alterations in dendritic cells, myeloid-derived suppressor cells, B and T cells, both in terms of cell count and activation status. The forthcoming results from tumour and microbiome analyses, and their integrated correlation, may help define prognostic and predictive signature with translational relevance in real world clinical practice.

Shifting to prognostic biomarkers, another article investigates the role of disulfidptosis, a novel cell death pathway, in NPC and HNSCC. Zhang et al. use GEO and TCGA datasets to show that the disulfidptosis-related gene SLC3A2 is overexpressed in these cancers, correlating with poor prognosis, reduced immune infiltration, and low expression of immune checkpoint genes. *In vitro* experiments on NPC cells confirm SLC3A2’s oncogenic effects on proliferation and migration, positioning it as a potential biomarker for adverse outcomes. This study underscores how multi-omics can link cell death mechanisms to immune evasion, opening avenues for targeted therapies disrupting disulfidptosis in HNC.

Understanding the molecular underpinnings of HNSCC progression is essential for biomarker development. Tang et al. employ high-throughput sequencing on HNSCC tissues and demonstrate that circRNA_036186 acts as a molecular sponge for miR-193b-3p, thereby upregulating 14-3-3ζ expression and promoting tumor cell proliferation, migration, and invasion. Elevated 14-3-3ζ correlates with poor survival and immune-related pathways, as validated through TCGA data and functional assays. By constructing a circRNA-miRNA-mRNA axis, this research illustrates the utility of transcriptomic profiling in uncovering non-coding RNA networks that drive HNSCC aggressiveness, suggesting circRNA_036186 as a possible diagnostic or therapeutic target.

Yang and Xie contribute to this mechanistic understanding by systematically reviewing long non-coding small nucleolar RNA host genes (SNHGs) in thyroid cancer, a related head and neck malignancy. Their comprehensive analysis reveals that SNHG family members regulate key cellular processes including proliferation, invasion, epithelial-mesenchymal transition, and drug resistance through competitive endogenous RNA networks. Notably, different SNHG members exhibit opposing functions, some acting as oncogenes (SNHG7, SNHG15, SNHG12, SNHG14) while others function as tumor suppressors (SNHG3, SNHG5). This highlights the complexity of lncRNA regulation, which may be influenced by the tissue-specific molecular environment and warrants further investigation.

All together, these articles demonstrate how multi-omics can illuminate immunological biomarkers in HNC, from systemic immune profiling and cell death pathways to non-coding RNAs, phase separation, and neoepitope prediction. Ongoing trials featuring perioperative checkpoint blockade, integration of organoid models for predictive testing, and implementation of longitudinal liquid biopsies represent critical steps in closing the gap between laboratory discoveries and improved patient outcomes. However, significant challenges remain on the path to precision immunotherapy for HNSCC. Response rates to immune checkpoint inhibitors remain heterogeneous, often limited by immune resistance mechanisms rooted in both the tumor and its microenvironment. Costs and complexity associated with multi-omic analyses can be prohibitive, requiring standardization and validation across diverse clinical settings.

By unveiling the molecular heterogeneity, immune landscapes, and regulatory networks that govern tumor behavior and treatment response, these studies provide a roadmap for identifying patients most likely to benefit from immunotherapy, developing rational combination strategies, and ultimately improving outcomes for HNC patients. The journey from multi-omics discovery to clinical implementation is lengthy and challenging, but the studies presented here represent critical steps toward realizing the promise of personalized immunotherapy in HNSCC.

